# Using a digital health intervention “INTERCEPT” to improve secondary prevention in coronary heart disease (CHD) patients: protocol for a mixed methods non-randomised feasibility study

**DOI:** 10.12688/hrbopenres.13781.1

**Published:** 2023-09-01

**Authors:** Irene Gibson, Catriona Jennings, Lis Neubeck, Marissa Corcoran, David Wood, Faisal Sharif, Lisa Hynes, Andrew W Murphy, Molly Byrne, John William McEvoy

**Affiliations:** 1 School of Medicine, University of Galway, Galway, Ireland; 2 National Institute for Prevention and Cardiovascular Health, Galway, Ireland; 3 School of Health and Social Care, Edinburgh Napier University, Edinburgh, UK; 4 Cardiology Department, St. James's Hospital, Dublin, Ireland; 5 Croí, West of Ireland Cardiac Foundation, Galway, Ireland; 6 Discipline of General Practice, University of Galway, Galway, Ireland; 7 Health Behaviour Change Research Group, University of Galway, Galway, Ireland

**Keywords:** Cardiovascular disease, digital health interventions, secondary prevention, feasibility, acceptability

## Abstract

**Background:** Digital health interventions (DHIs) are increasingly used for the secondary prevention of cardiovascular disease (CVD). The aim of this study is to determine the feasibility of “INTERCEPT”, a co-designed DHI developed to improve secondary prevention in hospitalised coronary heart disease patients (CHD).

**Methods:** This non-randomised feasibility study will be conducted using a mixed methods process evaluation with a sample of 40 patients in an acute hospital setting. Informed by behaviour change theory, the Intercept application (I-App) integrates a smartphone interface, health care professional portal, a fitness wearable and a blood pressure monitor. I-App is designed to support and motivate patients to set goals, self-monitor lifestyle and medical risk factors, and manage their medications, with the health care professional portal enabling monitoring and communication with patients. Using convenience sampling, eligible patients will be recruited in two phases, a pre-implementation phase and an implementation phase. During the pre-implementation phase participants will not immediately receive the I-App but will be invited to receive the I-App at 3 months follow-up. This will enable early learning about the processes of recruitment and conducting the assessment prior to full scale deployment of the I-App. During the implementation phase, participants will be invited to download the I-App to their smartphone prior to hospital discharge. Qualitative interviews will be conducted among a subset of patients and health care professionals to gain a greater insight into their experience of using the I-App. Primary outcomes will be assessed at baseline and 3-month follow-up. Using pre-defined feasibility criteria, including recruitment, retention and engagement rates, together with data on intervention acceptability, will determine the appropriateness of progressing to a definitive trial.

**Discussion:** This study will provide important insights to help inform the feasibility of conducting a definitive trial of “INTERCEPT” among coronary heart disease patients in a critical health care setting.

## Introduction

Cardiovascular disease (CVD) is one of the leading causes of death and disability globally, with approximately 40% of events occurring in patients with pre-existing coronary heart disease (CHD) ^1^. Comprehensive secondary prevention strategies, which involve behavioural lifestyle and medical risk factor management for patients with known CVD, can reduce CVD mortality, recurrent CVD hospital admissions, and improve overall quality of life ^2^. Yet, despite these well-established benefits, standards of secondary prevention are sub-optimal, with international data from the EuroAspire V survey and national data from the IAspire survey highlighting that the majority of CHD patients are not meeting the recommended secondary prevention lifestyle and risk factor targets ^3, 4^. Furthermore, while guidelines recommend that secondary prevention should start as early as possible following diagnosis ^5, 6^, referral to and uptake and accessibility of hospital-based, secondary prevention programmes such as cardiac rehabilitation(CR), remains persistently poor ^4, 7^. Therefore, to maximise uptake and participation rates, there is a need to look beyond traditional hospital-based CR programmes to more innovative, patient centred, delivery models, which focus on early initiation of prevention ideally within two weeks of the patients index event or hospitalisation ^8^. Evidence suggests that early initiation of prevention during this critical time point, when the patient is more likely to be motivated and engaged leads to greater uptake and adherence of prevention and rehabilitation programmes ^9, 10^.

Accelerated by the coronavirus disease 2019 (COVID-19) pandemic, there is increased recognition of the potential of DHIs to transform preventive care, with evidence suggesting improved CVD risk factor control, health related quality of life, medication adherence, enhanced self-management and shared decision making among patients with coronary heart disease ^11– 13^. Consequently, various International organisations such as the World Heart Federation, American Heart Association and the European Society of Cardiology recommend the use of digital interventions for the prevention and management of CVD ^14– 17^. Digital health is an evolving area with technologies encompassing electronic decision support tools, eHealth (electronic health records) artificial intelligence, machine learning, telemedicine and mHealth (smart phone apps, wearables, text messaging). With 70% of the world’s population using a smart phone ^18^, smart phone applications are an obvious choice to increase the reach of secondary prevention interventions. However, despite their exponential growth, uptake and usage of health apps by patients is low ^19^ and there have been limited studies evaluating health apps in critical care settings such as the coronary care unit ^20^. Additional challenges of DHIs are that, the majority of apps are designed with minimal input from target end users, the degree to which they follow evidence-based guidelines is unclear, and there is lack of understanding of the systems required to support implementation and scalability of these apps ^11, 19^.

To address these challenges, we have developed a digital intervention known as “INTERCEPT”, which aims to improve secondary prevention in CHD patients. Responding to the need for early initiation of prevention following an index event, the INTERCEPT intervention will be introduced to the patient at the time of their acute hospitalisation and before discharge. The intervention includes, (1) a smart phone app which aims to support and motivate patients to achieve a healthy lifestyle, manage their CVD risk factors to target, and improve adherence with cardio protective medications and (2) a web-based healthcare professional (HCP) portal, which will support remote monitoring of lifestyle, medical risk factor and medications and facilitates direct communication with the patient by a specialist cardiovascular nurse. To enable self-monitoring in real time INTERCEPT integrates with a fitness wearable and a blood pressure monitor.

To optimise the INTERCEPT application (hereafter called ‘I-App’) and its impact in terms of improving the standards of secondary CVD care, the development of I-App has been guided by the Medical Research Council (MRC) guidelines for the development and evaluation of complex interventions ^21^. To ensure it meets the needs of the end user, I-App and the protocol for this feasibility study has been co-designed with key stakeholders including patients, healthcare professionals and software developers. Our next step in the intervention development process is to examine the feasibility of the I-App in the real-world clinical setting. Acknowledging that feasibility is an overarching concept ^22^, specific feasibility domains such as the acceptability and usability of the intervention, recruitment capability, retention of study participants and study assessment procedures, will be examined, through our study objectives ^23^.

### Aims & objectives

The overall aim of this study is to examine the feasibility of the INTERCEPT digital intervention, to help inform (a) further refinement of the intervention, and (b) to determine the feasibility of a definitive randomized controlled trial (RCT).

The primary objectives are to assess:

- The acceptability of the I-App intervention among patients and health care professionals through semi-structured interviews and by examining recruitment and retention rates. Acceptability is defined as the extent to which people delivering or receiving a health care intervention consider it appropriate based on anticipated or experienced cognitive and emotional responses to the intervention ^24^.
- Engagement with the I-App by examining the extent (e.g. amount, frequency, duration, depth) of usage among patients.

The secondary objectives are to:

- Assess the feasibility of the study methods, by examining recruitment and retention rates assessment and data collection procedures and analysis methods
- Obtain preliminary data of the potential association of the I-App with improved lifestyle, psychosocial and medical risk factors for CVD and adherence with cardio protective medications at 3 months

## Methods

### Study design

This is a non-randomised, pilot feasibility study, with an embedded process evaluation. Process evaluations aim to explain how complex interventions work, providing information on implementation process, the mechanisms of change (how does the intervention produce change) and contextual factors, all of which may influence study outcomes ^25^. While the purpose of a process evaluation varies depending on the stage of intervention development, for this study it will play an important role in understanding the feasibility and acceptability of the I-App and optimising its design in preparation for a larger scale effectiveness trial ^21, 25^. We will also deploy a mixed methods approach for this feasibility study, i.e., combining the strengths of both quantitative and qualitative methods. This will maximise what can be learnt from our feasibility study and thus inform a robust decision about next steps ^23^. Given potential uncertainties around the feasibility of recruiting participants and deploying a DHI in a critical care setting such as the Coronary Care Unit (CCU) or Cardiothoracic Unit (CTU), this study will be conducted in 2 phases, a pre- implementation phase and an implementation phase. The pre-implementation phase will be one month long and we anticipate enrolling 15 participants during this time. While, participants in this phase will undergo the study assessment procedures, they will not immediately receive the I-App, but they will be invited to receive the I-App at 3 months follow-up. This will enable us to learn about the processes of recruitment and conducting the assessment prior to full scale technical deployment of the I-App, which potentially will bring additional challenges.

The implementation phase will be two months long and we anticipate enrolling 25 participants during this time. Acknowledging that this is a non-randomised feasibility study, the CONSORT extension for reporting pilot randomised controlled trials will be used to guide reporting where applicable ^26^

### Study population & recruitment

The planned inclusion criteria are patients (≥18years) with a diagnosis of CHD. This includes acute coronary syndrome patients or those who have had elective percutaneous transluminal coronary angioplasty or coronary artery by-pass surgery. Patients must have a smart phone or tablet to enable download of the app, have access to email on this device and be able to provide written consent in English. The exclusion criteria are patients who are clinically unstable and are not planned for discharge home.

Using convenience sampling eligible patients will be recruited to the study through the cardiology department (coronary care and cardiothoracic units) at University Hospital Galway. Nurses working in these units will inform patients of the study using an information flyer. Enrollment to the control group (pre-implementation phase) and intervention group (implementation phase) will be based on consecutive ACS patients admitted to hospital in defined date periods.

As this is a feasibility study no formal power calculations are required to determine sample size. Recommendations on the appropriate sample size for these types of studies vary greatly from 10–12 participants per group to between 24–50 per group ^27^. We have chosen a sample size of 40 (15 pre-implementation, 25 implementation) based on similar studies and practical time frames ^27, 28^. For the qualitative semi-structured interviews, purposive sampling (i.e. the deliberate choice of a participant due to particular qualities they possess) will be used to recruit 10–15 patients and 6–8 health care professionals and researchers. The concept of information power, will be used to guide final sample size. Information power indicates that the more information the sample holds, relevant to the actual study the lower the amount of participants required ^29^.

### I-App intervention

I-App is a complex intervention, which aims to support self-management and motivate patients to achieve a healthy lifestyle, manage CVD risk factors, and improve adherence with cardio protective medications. It includes a web-based, health care professional portal and a smart phone app, which integrates with a fitness wearable and blood pressure monitor. I-App has been co-designed by a core team of health care professionals (nurse specialists, physiotherapists, dietitians, psychologists, cardiologists, and a pharmacist) researchers, software developers and patients from the Croí (a heart and stroke patient organisation) public and patient involvement (PPI) panel. An iterative and participatory approach to the design process was adopted using online workshops conducted over a 12-month period. This involved identification of the guiding principles, content and design features, developing a working prototype of the app, followed by a beta version which was pilot tested among the project team and reviewed for clarity of language, ease of navigation and functionality. To anticipate and interpret intervention usage, user acceptance testing with patients who had a recent cardiac event (<2 years) was conducted. 

A full description of the intervention components are presented in *Extended data* ^30^. In summary, these include: tailored goal setting to motivate and support healthy lifestyle changes; a health tracker to support self-monitoring of physical activity, mood, healthy eating, medications, blood pressure, cholesterol and glucose levels; educational resources to increase knowledge and awareness of healthy lifestyle changes and adherence with cardio-protective medications and notifications to prompt engagement with the I-App. The HCP portal is designed to support remote monitoring and communication with patients. Through informed consent, a CVD Nurse Specialist will monitor patient engagement with the I-App through the portal dashboard. This data includes tracking of lifestyle and medical risk factors, goal setting and use of medication reminders. As the I-App has been designed as a self-management tool the nurse will only initiate contact with the patient, when self-reported outcomes are outside guideline recommended targets, for example if blood pressure is high. In line with a protocol, the patient will then be advised to follow-up with their GP or Cardiologist.

Given the strong focus of the I-App in supporting and changing health behaviours its development has been informed by social cognitive theory ^31^ and select behaviour change techniques from the taxonomy of behaviour change techniques (BCTs) ^32^. An illustration of how the components and features of the intervention are aligned with behavioural change techniques, proposed mechanism of actions and outcomes is presented in the I-App logic model (can be reviewed in *Extended data ^14^*).

### Study procedures

All patients will undergo a baseline assessment prior to hospital discharge which will include: demographics (including age, sex, ethnicity, education); medical history; measurement of health behaviours (physical activity, diet), weight, height and waist, blood pressure, lipids, blood glucose and HbA1c, digital health literacy (eHealth literacy Scale) and health related quality of life.

During the implementation phase patients will be invited to download the I-App to their smartphone. They will be supported by a study nurse who will explain how to use the I-App. Participants will also be given a hard copy user manual, which will provide detailed instructions for using I-App. Patients will receive a blood pressure monitor (Withings BPM), a fitness wearable (Withings Pulse HR) and weighing scales, all of which will integrate with the I-App using luetooth technology. Together with the I-App, this equipment will support the patients in monitoring and tracking their lifestyle and medical risk factors. With their informed consent, the nurse will track the data they record on the I-App through the web-based nurse portal and will provide appropriate prompts to seek professional help, as required, through their own general practitioner.

**Figure 1.**
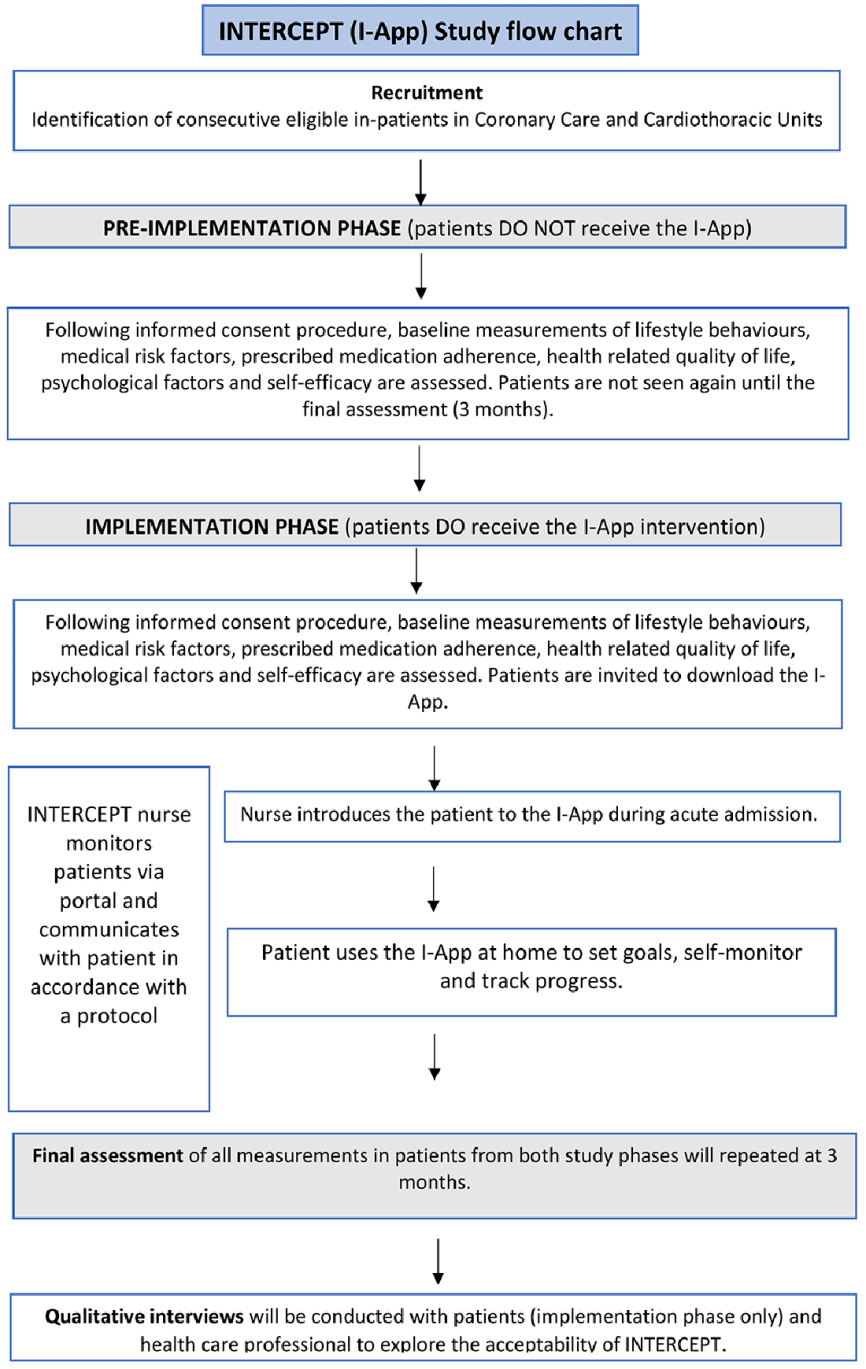
Intercept study flow.

A second in person follow up assessment will be conducted 3 months after the first assessment, following the patient’s discharge from hospital. At this assessment, patients who were enrolled during the pre-implementation phase will be offered access to the I-App and the supporting devices (Fitness wearable, blood pressure monitor and weighing scales). Figure 1 illustrates the flow of participants throughout the study.

To explore the acceptability of I-App, semi-structured interviews will take place with a subset of patients and health care professionals at 3-month follow-up. The interview guides can be reviewed in *Extended data ^14^*. These interviews will provide an in-depth understanding of their perspectives of the I-App, exploring attitudes, usability and satisfaction. Furthermore, the interviews will help explore the relationship between mechanisms of behaviour change, the implementation of I-App and the context within which it is being implemented. Using a convergent approach both quantitative and qualitative data will be collected during the same stage of the research and will be merged to create a more comprehensive interpretation of the data ^23^. However, if implementation barriers arise during the study, for example if participants withdraw from using the I-App soon after receiving it, the timing of the interviews will be reviewed, to ensure data capture. Mixed methods guidance recommends this approach as it helps to inform refinement to the recruitment strategy ^23^. To ensure quality of reported results, the consolidated criteria for reporting qualitative research (COREQ) will be used ^33^.

### Outcomes

Outcomes will be assessed at baseline (time point 1, T1) and at 3 months (time point 2, T2). Table 1 provides a summary of the outcomes, associated measures and time points, which correspond to the primary and secondary study objectives. 

**Table 1.** Summary of outcome measures.

Objectives	Measures/approaches	Time points
Primary		
Acceptability of I-App to patients and healthcare professionals	• No of eligible patients invited to participate in the study • No of patients who enrolled in the study • % of enrolled patients who attended 3 month follow-up • Semi structured interviews guided by the theoretical framework of acceptability *	T1 T1 T2 T2
Engagement and usability of I-App among patients *	• Using web analytics, app usage data including logs of interactions (date and time of use, modules viewed and time spent on them) and user-entered data (for example blood pressure readings or goals set) will be measured. • System Usability Scale (SUS) ^35^. This scale provides a measure of the person’s subjective perceptions of the usability of a system over a short period of time. It assesses the components of usability, effectiveness and efficiency and satisfaction according to the user and context of use.	T2 T2
Secondary		
Lifestyle	**Smoking cessation**: self-reported and validated by breath carbon monoxide using the Bedfont Smokerlyser (Micro+) **Mediterranean Diet Score**: self-reported using the 14 item Mediterranean Diet Questionnaire ^36^ **Physical Activity and exercise**: International Physical Activity Questionnaire (IPAQ) short form **Weight**: • Change in weight in those overweight or obese (as defined by body mass index) at hospital admission using SECA 701 digital scales • Change in waist reduction in those centrally obese (waist circumference) at hospital admission using a metal tape measure	T1 & T2 T1 & T2 T1 & T2 T1 & T2
Medical risk factors	• % Blood pressure < 130/80mmHg and < 140/80 mmHg • % Low-density lipoprotein (LDL) cholesterol< 1.4 mmol/l • % HbA1c < 53mmol/mol • % Fasting blood glucose ≤ 6 mmol/l	T1 & T2 T1 & T2
Cardio protective medications	Beliefs about and adherence with medications will be measured using the Medication Adherence Report Scale(MARS-5) ^37^	T1 & T2
Psychosocial	• Health related quality of life (HRQoL) using HeartQoL ^38^ • Anxiety and depression using the Hospital Anxiety and Depression Scale (HADS) ^39^ • General Self-Efficacy Scale ^40^	T1 & T2 T1 & T2 T1 & T2
Feasibility of a definitive trial of the I-APP	Acceptability and suitability of the study procedures will be assessed through semi-structured interviews with both patients and health care professionals	T2

*Acceptability, usability and engagement of the I-App will be measured among participants in the implementation phase only.

### Progression criteria

Progression to a definitive trial will be determined by pre-defined “Stop/Amend/Go progression criteria ^34^ which have been developed through study team consensus. Beyond examining quantitative measures of recruitment and retention we have incorporated qualitative methods to ensure a more comprehensive understanding of the feasibility of implementing the I-App in a critical care setting is obtained. These criteria are outlined in Table 2. In addition, the decision to progress will be informed by the acceptability of the I-App to both patients and health care professionals and suggested refinements to the intervention will be reviewed prior to progression.

**Table 2.** Intercept progression criteria.

	Go - proceed with RCT	Amend – proceed with changes	Stop - do not proceed unless changes are possible.
Feasibility of patient recruitment.	>75% of the target sample (n=40) size are recruited in four months.	30–74% of the sample size are recruited in four months.	<30% of the sample size are recruited in four months.
Feasibility of patient retention	>80% of enrolled patients attend 3 month follow-up.	60–80% of enrolled patients attend 3 month follow-up	<60% of enrolled patients attend 3 month follow-up.
Feasibility of intervention implementation	Delivery of intervention judged strongly feasible by qualitative data.	Delivery of intervention judged possibly feasible by qualitative data.	Delivery of intervention judged not feasible by qualitative data.

### Data analysis

***Qualitative analysis*.** Qualitative data will be initially analysed using thematic analysis, following which the Theoretical Framework of Acceptability (TFA) will be deductively applied. The TFA is designed to assess acceptability across seven constructs: affective attitude, burden, ethicality, intervention coherence, opportunity costs, perceived effectiveness, and self-efficacy and is recommended for use in feasibility studies ^24^. A definition of these constructs is provided in Table 3. Analysis will be supported by use of NVivo, a qualitative data analysis software package. Two members of the research team will code the data and will assess the information power of the sample. These two individuals, together with the study team including the PPI group, will work collaboratively to interpret the findings.

**Table 3.** Theoretical Framework of Acceptability Constructs ^24^.

Theoretical Framework of Acceptability (TFA) Constructs	Definition
Affective attitude	How an individual feels about the intervention, after taking part.
Burden	The amount of effort that was required to participate in the intervention.
Ethicality	The extent to which the intervention has good fit with an individual’s value system.
Opportunity costs	The benefits, profits or values that were given up to engage in the intervention.
Perceived effectiveness	The extent to which the intervention is perceived to have achieved its intended purpose.
Self-efficacy	The participant's confidence that they can perform the behaviour(s) required to participate in the intervention.
Intervention coherence	The extent to which the participant understands the intervention and how it works.

***Quantitative analysis*.** Descriptive statistics will be used to report on baseline demographics, clinical data, I-App usability and usage data. Continuous variables will be presented as means with standard deviations and categorical variables in absolute frequencies with percentages. As this is a feasibility study, we do not aim to assess statistical significance between the pre-implementation and implementation phase. However to obtain preliminary data on the possible association of the I-App with improved lifestyle, psychosocial and medical risk factors for CVD at 3 months we will examine outcomes between baseline and end of study assessment. Changes in categorical variables will be assessed by the McNemar test and the paired t-test or Mann-Whitney will be used for continuous variables. Recruitment and retention rates will be reported on and presented using the CONSORT flow diagram ^41^. All statistical analysis will be conducted using Stata. 16.

### Ethics

Ethical approval for this study was granted by the Clinical Research Ethics Committee at Galway University Hospitals (C.A. 2913) on the 16 ^th^ of March 2023. Informed consent will be obtained following explanation of the study and the provision of the patient information leaflet. All participants will be informed of their right to withdraw from the study at any time without giving a reason.

### Public and patient involvement

Patient and Public Involvement (PPI) from the Croí (heart and stroke patient organisation) PPI panel has been embedded in this study from the outset. The PPI panel, which includes 5 contributors (4 female) with lived experience of CVD have been involved in the co-design of the Intercept intervention, proving input across all stages of the design process. For this specific study they attended two 1.5 hour meetings (one online and one in person) providing advice on the recruitment strategy, technology deployment and the study materials including the study flyer, patient information leaflet, consent form and topic guides for the qualitative interviews. It is anticipated that future contributions will include supporting data analysis of the qualitative interviews, interpreting the results of these interviews and advising on communication and dissemination of research outcomes.

### Data management

All data will be managed in line with GDPR requirements: data minimisation, storage limitation, transparency, integrity and confidentiality. A data protection impact assessment has been prepared and will be reviewed before the study commences, and as necessary over the course of the study.

### Plans for dissemination of the study outcomes

A knowledge exchange and dissemination plan has been developed with key project stakeholders including the Croí PPI panel. Accordingly, study outcomes will be published in a peer-reviewed journal and will be presented at relevant national and international conferences. Given the relevance of the study to national priorities regarding chronic disease prevention, findings will be shared with the Health Service Executive (HSE) leads for the Integrated Care Programme for the Prevention and Management of Chronic Disease, the National Heart Programme and the Digital Transformation team. Outcomes will be shared with other key stakeholders including patient organisations such as Croí, hospital and community cardiology and rehabilitation teams, study participants and members of the public.

## Study status

At the time of publication, recruitment to this study had commenced (June 2023).

## Discussion

This study protocol describes the methods used to assess the feasibility of a trial of “INTERCEPT” a mobile Health app linked to a HCP portal and wearable technology to improve secondary prevention in CHD patients. Guidelines recommend that secondary prevention should start as early as possible after a cardiac event ^5, 6^, in reality, referral and uptake of secondary prevention interventions such as CR remains persistently poor and often is very delayed. The I-App aims to bridge this important care gap by providing CHD patients with early access to a digital secondary prevention intervention at the time of their diagnosis and prior to their discharge from hospital. By examining the acceptability and usability of the I-App among a sample of these patients, it will enable us to further refine the I-App intervention to optimise its acceptability, use and effectiveness prior to moving to a definitive RCT. Moreover, by applying the pre-defined “Stop/Amend/Go progression criteria this will provide transparent and objective justification on the appropriateness of moving to a larger trial.

There are some potential challenges associated with this study. Firstly, the time frame for recruitment is short as many CHD patients are discharged back to the referring hospital within 24 hours or home within 48 hours. This offers a limited window to conduct the initial assessment and to support the patient with downloading the I-App. Through engagement with key stakeholders, including CCU/CTU nurses and cardiology staff, we have attempted to address this challenge by refining the recruitment strategy, baseline assessment procedures and including a pre-implementation phase, which may lead to subsequent refinements. Secondly, the field-based researchers and healthcare professionals involved in this study have limited experience in digital health intervention research and therefore may encounter challenges related to the technical aspects of deploying the I-App. To overcome these challenges, we have collaboratively developed a guidance document, provided hands-on training, and have put in place a technical support helpline. 

As the majority of DHI research for the secondary prevention of CVD is conducted in outpatient settings, this study will contribute to the evidence base related to the feasibility of introducing a DHI to CHD patients in a critical care setting at the time of diagnosis and before hospital discharge. Furthermore, through the application of a mixed methods approach, a comprehensive, nuanced and context specific understanding of the potential feasibility and acceptability issues will be generated, which will help inform decisions regarding a definitive trial of INTERCEPT. 

## Data Availability

No underlying data are associated with this article. Open Science Framework: INTERCEPT. https://doi.org/10.17605/OSF.IO/85CV4 ^30^. This project contains the following extended data: Consent form_ healthcare professional and researcher Consent form–Patient V2 INTERCEPT Intervention components INTERCEPT logic model Interview topic guides PIL-Healthcare professional and researcher PIL-Patient V2 Data are available under the terms of the Creative Commons Attribution 4.0 International license (CC-BY 4.0).
