# Supplementary material for: Using a digital health intervention “INTERCEPT” to improve secondary prevention in coronary heart disease (CHD) patients: protocol for a mixed methods non-randomised feasibility study
Source: HRB Open Res. 2025 Jan 31;6:43. Originally published 2023 Sep 1. [Version 2] doi: 10.12688/hrbopenres.13781.2 (PMC10897504; doi:10.12688/hrbopenres.13781.2)
Supplement: Peer review history

# Reviewer report: Reviewer response for version 2

**DOI:** 10.21956/hrbopenres.15410.r45222

**Published:** 2025-02-06

**Authors:** Stefan Tino Kulnik

**Affiliations:** 1 Ludwig Boltzmann Institute for Digital Health and Prevention, Lindhofstrasse 22, 5020, Salzburg,, Austria

I thank the authors for incorporating my suggestions.

Is the study design appropriate for the research question?

Yes

Is the rationale for, and objectives of, the study clearly described?

Yes

Are sufficient details of the methods provided to allow replication by others?

Yes

Are the datasets clearly presented in a useable and accessible format?

Not applicable

Reviewer Expertise:

Digital Health (dHealth), Mobile Health (mHealth), Cardiac Rehabilitation, Secondary Prevention of Cardiovascular Disease, Physiotherapy, Complex Interventions

I confirm that I have read this submission and believe that I have an appropriate level of expertise to confirm that it is of an acceptable scientific standard.

# Reviewer report: Reviewer response for version 1

**DOI:** 10.21956/hrbopenres.15075.r37825

**Published:** 2024-02-26

**Authors:** Stefan Tino Kulnik

**Affiliations:** 1 Ludwig Boltzmann Institute for Digital Health and Prevention, Lindhofstrasse 22, 5020, Salzburg,, Austria

Peer review

 Using a digital health intervention “INTERCEPT” to improve secondary prevention in coronary heart disease (CHD) patients: protocol for a mixed methods non-randomised feasibility study

 Gibson I, Jennings C, Neubeck L, Corcoran M, Wood D, Sharif F, Hynes L, Murphy AW et al.

 Thank you for inviting my peer review of this study protocol, describing a non-randomised pilot feasibility study with embedded process evaluation. The authors have developed a digital health intervention (INTERCEPT, I-App) to support patients with coronary heart disease in the secondary prevention and self-management of their cardiovascular condition.

 This is a well-written and comprehensive study protocol, incorporating relevant methodological literature (MRC framework for complex interventions, behaviour change techniques taxonomy) and reporting standards (CONSORT, COREQ), and providing additional study documentation in the online extended data repository (Open Science Framework).

 I only have very minor comments for the authors‘ consideration, which are all intended to support the clarity of the report and to add some details for readers‘ information and completeness:

 In the abstract, I suggest using the same wording to describe the study design as in the main text, i.e., a non-randomised, pilot feasibility study with embedded process evaluation (as opposed to „…will be conducted using a mixed methods process evaluation“). Also in the abstract, the description of the timing/duration of the pre-implementation and implementation phases is not so clear – perhaps this can be re-worded.

 Introduction: „eHealth (electronic health records)“ could be read as eHealth = electronic health records, while eHealth encompasses a wider range of modalities/interventions.

 Aims & objectives: Recruitment and retention rates are included under the primary objective „to assess the acceptability of the I-App intervention“, but recruitment and retention better fit with the secondary objective „to assess the feasibility of the study methods“ (which also describes recruitment and retentention). To assess the acceptability of the I-App intervention, perhaps the wording „uptake and engagement with the I-App“ would be preferable? I would suggest the same for Table 1, where the first three bullet points (number eligible, number enrolled, number completing follow-up) better fit with the last objective „Feasibility of a definitive trial“.

 Recruitment: I’m unsure that this is „convenience sampling“, since, as I understand it, all consecutive patients on the respective wards will be systematically screened for eligibility. Convenience sampling to me would describe a less systematic approach.

 Purposive sampling for qualitative interviews: Could the authors add examples for particular selection criteria, e.g., representation of both genders, surgical and non-surgical treatment, good and poor engagement with the I-App, etc. Specify „The concept of information power will be used to guide final sample size for qualitative interviews.“

 I-App intervention: I would assume that the I-App intervention has been developed specifically for the Irish healthcare context. Could the authors add some detail, e.g., what context was the intervention developed for, from which contexts were the co-design contributers, and what were the dates (year/s) for the development phase.

 Study procedures: For completeness, please specify and reference the data collection instruments for physical activity, diet, digital health literacy and quality of life here in the main text. Please reference IPAQ in table 1; typo „luetooth technology“; please add how frequently the nurse will track I-App users‘ data, e.g. weekly, daily, etc.; For how long will patients from the pre-implementation group be allowed to use the I-App after the 3-months follow-up? Specify „ audio-recorded semi-structured interviews“; specify „To ensure quality of reported qualitative results, the consolidated…“

 Data analysis: State whether interview recordings will be transcribed.

 Figure 1: I wonder how the timing of the pre-implementation phase and implementation phase could be visualised, so that the flow diagram reads unambiguously. At the moment, readers might think that this is one and the same cohort of patients progressing through both phases. Perhaps 2 parallel strands/columns for the 2 phases, both leading to the final assessment box, and staggered to indicate the different recruitment periods? It would be helpful to state „first month of recruitment“ for the pre-implementation phase, and „second and third months of recruitement“ for the implementation phase; and to include T1 and T2 in the flow diagram.  

 Table 2, row „Feasibility of patient recruitment“: in three months rather than in four months?

 Discussion: Sentence „Guidelines recommend that…“ is missing a word; „Stop/Amend/Go is missing a quotation mark.

 I noticed that acronyms are sometimes not spelled out at first use (DHI, GDPR), and sometimes spelled out after they have already been defined (PPI, DHI).

 Extended data: In the description of the I-App, it would be helpful to provide screenshots for each view/function. With regard to the healthcare professionals‘ dashboard, please describe whether healthcare professionals can communicate with patients through the app („in-app“) or via communication channels outside of the app (telephone, etc.). 

 In the description of the logic model, it would be helpful to add a figure legend, including an explanation of the numbering of the behaviour change techniques (presumably these refer to categories of the taxonomy of behaviour change techniques). I noticed that the outcomes in the logic model do not include reduction in the occurrence of cardiovascular events and re-hospitalisation or reduction in mortality – was this a deliberate decision (and why)?

Is the study design appropriate for the research question?

Yes

Is the rationale for, and objectives of, the study clearly described?

Yes

Are sufficient details of the methods provided to allow replication by others?

Yes

Are the datasets clearly presented in a useable and accessible format?

Not applicable

Reviewer Expertise:

Digital Health (dHealth), Mobile Health (mHealth), Cardiac Rehabilitation, Secondary Prevention of Cardiovascular Disease, Physiotherapy, Complex Interventions

I confirm that I have read this submission and believe that I have an appropriate level of expertise to confirm that it is of an acceptable scientific standard.

# Response

**Published:** 2024-11-21

**Authors:** Irene Gibson

**Affiliations:** School of Medicine, University of Galway, Galway, Ireland

We thank the reviewer for taking the time to provide such a thorough and helpful review of the manuscript. The comments have been most useful and we are confident that our revised submission has improved by addressing them. A point-by-point response to each of these comments is provided as follows:

  

1. **In the abstract, I suggest using the same wording to describe the study design as in the main text, i.e., a non-randomised, pilot feasibility study with embedded process evaluation (as opposed to „…will be conducted using a mixed methods process evaluation“). Also in the abstract, the description of the timing/duration of the pre-implementation and implementation phases is not so clear – perhaps this can be re-worded.**

We have revised the abstract in line with these suggestions, thank you.

  

1. **Introduction: „eHealth (electronic health records)“ could be read as eHealth = electronic health records, while eHealth encompasses a wider range of modalities/interventions.**

To avoid potential confusion, we have removed reference to electronic health records.

  

1. **Aims & objectives: Recruitment and retention rates are included under the primary objective „to assess the acceptability of the I-App intervention“, but recruitment and retention better fit with the secondary objective „to assess the feasibility of the study methods“ (which also describes recruitment and retentention). To assess the acceptability of the I-App intervention, perhaps the wording „uptake and engagement with the I-App“ would be preferable? I would suggest the same for Table 1, where the first three bullet points (number eligible, number enrolled, number completing follow-up) better fit with the last objective „Feasibility of a definitive trial“.**

We agree, recruitment and retention rates align better with the secondary objectives of the study “assessing the feasibility of study methods”. We have updated the objectives and Table 1 to reflect this change.

  

1. **Recruitment: I’m unsure that this is „convenience sampling“, since, as I understand it, all consecutive patients on the respective wards will be systematically screened for eligibility. Convenience sampling to me would describe a less systematic approach.**

Thank you for highlighting this important point. We have replaced all references to convenience sampling with consecutive sampling, to accurately reflect our approach to sampling.

  

1. **Purposive sampling for qualitative interviews: Could the authors add examples for particular selection criteria, e.g., representation of both genders, surgical and non-surgical treatment, good and poor engagement with the I-App, etc. Specify „The concept of information power will be used to guide final sample size for qualitative interviews.“**

This is a great suggestion. We have now elaborated on the selection criteria for the qualitative patient interviews, which includes reference to all of the above points. 

  

1. **I-App intervention: I would assume that the I-App intervention has been developed specifically for the Irish healthcare context. Could the authors add some detail, e.g., what context was the intervention developed for, from which contexts were the co-design contributers, and what were the dates (year/s) for the development phase.**

Thanks for another helpful suggestion. These additional details, including the origins of INTERCEPT have now been provided.

  

1. **Study procedures: For completeness, please specify and reference the data collection instruments for physical activity, diet, digital health literacy and quality of life here in the main text. Please reference IPAQ in table 1; typo „luetooth technology“; please add how frequently the nurse will track I-App users‘ data, e.g. weekly, daily, etc.; For how long will patients from the pre-implementation group be allowed to use the I-App after the 3-months follow-up? Specify „audio-recorded semi-structured interviews“; specify „To ensure quality of reported qualitative results, the consolidated…“**

While the data collection instruments were outlined in table 1, to respond to this comment and improve the clarity of the paper we have updated the study procedures text to include this information. In addition, we provide details on the frequency of monitoring the HCP portal and INTERCEPT usage after the 3 month follow-up. Suggested text changes to offer greater clarity on the semi-structured interviews and use of the COREQ have also been included and the typo has been corrected.

  

1. **Data analysis: State whether interview recordings will be transcribed.**

Thanks, we have added this additional detail in under qualitative analysis.

  

1. **Figure 1: I wonder how the timing of the pre-implementation phase and implementation phase could be visualised, so that the flow diagram reads unambiguously. At the moment, readers might think that this is one and the same cohort of patients progressing through both phases. Perhaps 2 parallel strands/columns for the 2 phases, both leading to the final assessment box, and staggered to indicate the different recruitment periods? It would be helpful to state „first month of recruitment“ for the pre-implementation phase, and „second and third months of recruitement“ for the implementation phase; and to include T1 and T2 in the flow diagram.  **

This is an excellent suggestion and we have now revised the flow diagram to delineate between the pre-implementation and implementation phases.  

  

1. **Table 2, row „Feasibility of patient recruitment“: in three months rather than in four months?**

The table has been updated to reflect the 3-month follow-up period.

  

1. **Discussion: Sentence „Guidelines recommend that…“ is missing a word; „Stop/Amend/Go is missing a quotation mark.**

Thank you for highlighting these grammatical errors, which have now been corrected.

  

1. **I noticed that acronyms are sometimes not spelled out at first use (DHI, GDPR), and sometimes spelled out after they have already been defined (PPI, DHI).**

These grammatical errors have been corrected, throughout the manuscript

  

1. **Extended data: In the description of the I-App, it would be helpful to provide screenshots for each view/function. With regard to the healthcare professionals‘ dashboard, please describe whether healthcare professionals can communicate with patients through the app („in-app“) or via communication channels outside of the app (telephone, etc.). **

We welcome this great suggestion, however we deliberately did not include screen shots as they have been incorporated in a separate publication, which describes the co-design and development of INTERCEPT. This publication is currently under review. We have provided additional detail on health care professional communication in the extended data.

  

1. **In the description of the logic model, it would be helpful to add a figure legend, including an explanation of the numbering of the behaviour change techniques (presumably these refer to categories of the taxonomy of behaviour change techniques). I noticed that the outcomes in the logic model do not include reduction in the occurrence of cardiovascular events and re-hospitalisation or reduction in mortality – was this a deliberate decision (and why)?**

Thanks again for your very helpful attention to detail. For the logic model we have now added a figure legend, which offers greater clarity on the BCTs.  You raise an important point regarding outcomes, however the behavioural and biomedical outcomes included in the logic model relate to the expected mechanisms of action of the INTERCEPT components. There is significant evidence to show that these outcomes can significantly reduce the incidence of repeat CV events and death. For next step evaluation of effectiveness, it is likely that we will assess 30-day hospital readmissions, however we anticipate that a much longer-term follow-up, would be required to observe meaningful reductions in repeat CVD events and overall mortality.

# Reviewer report: Reviewer response for version 1

**DOI:** 10.21956/hrbopenres.15075.r36520

**Published:** 2023-10-12

**Authors:** Geraldine Lee

**Affiliations:** 1 King's College London, London, England, UK

A well thought study protocol using a mixed methods approach. The protocol is detailed and including digital technology (i.e. Smartphone) along with HCP portal with BP monitor and fitness monitor is a novel approach. The phases with pre-implementation and implementation are justified and recruited numbers realistic. The only issue is that when patients experience an acute event, they are often overwhelmed and may decline the study. It is important that a close eye is kept on numbers declining the study. 

 The inclusion criteria are acceptable and primary objectives are appropriate. The goal setting for participants is good and it will be interesting to see the level of engagement and whether participants maintain engagement for the duration of the study. The interviews with HCPs and participants will be beneficial in identifying issues that would have potentially been missed by using a purely quantitative approach. 

 A well written protocol that will provide useful data on early engagement with patients after an acute CVD event.

Is the study design appropriate for the research question?

Yes

Is the rationale for, and objectives of, the study clearly described?

Yes

Are sufficient details of the methods provided to allow replication by others?

Yes

Are the datasets clearly presented in a useable and accessible format?

Not applicable

Reviewer Expertise:

Cardiovascular disease, risk factor modification, behaviour change in chronic diseases, nursing, mixed methods, lived experience.

I confirm that I have read this submission and believe that I have an appropriate level of expertise to confirm that it is of an acceptable scientific standard.
